# The Diagnosis of Pyothorax1Read before the Buffalo Academy of Medicine, January 4, 1898.

**Published:** 1898-07

**Authors:** Eugene A. Smith

**Affiliations:** Buffalo, N. Y., Professor of anatomy and the principles of surgery at Niagara University Medical College


### THE DIAGNOSIS OF PYOTHORAX.[1]

By EUGENE A. SMITH, M. D., Buffalo, N. Y.,
Professor of anatomy and the principles of surgery at Niagara University Medical College.

IN THE course of most diseases there is a time when the casual observer can hazard a close guess as to their nature and a superficial clinical examination is certain to make the diagnosis. The obscurity to the nature of a disease in its early stages lies in the mature development of its characteristic group of symptoms, while later in the case it is due to the additional features which are caused by the development of complications. In cases of pyothorax which are fairly established and later, if complications are not present, the diagnosis is not difficult. But early in this disease and later in some of its more unusual forms and in the presence of complications the diagnosis is far from easy.

Judging from my experience, most cases of pyothorax which reach the surgeon for operation do so when they have progressed much nearer extreme danger or impending death undiagnosticated than should be necessary. Quite often the signs and symptoms upon which to base a diagnosis are ready for the clinician many hours or days before they are detected. Such delay cannot fail to influence the patient's chances most unfavorably. The operative treatment of empyema results so favorably when the diagnosis is made early and prompt thoracotomy is performed that the urgency of an early recognition of the condition is to be emphasised, more especially when the deplorable results of the old aspiration methods of treatment obtained no later than fifteen or twenty years ago, are contrasted with the brilliant results of thoracotomy as performed today.

In children, especially, delay in the diagnosis of pyothorax most often occurs. The struggles and cries of the child during physical exploration, the tendency to explain a slow convalescence in a case of pneumonia or pleuro-pneumonia in some other way than as due to pus in the pleural cavity, and the hesitancy to interpret the physical signs as indicating the need of exploratory needle puncture, seem to be the factors delaying the determination of the diagnosis.

1. Read before the Buffalo Academy of Medicine, January 4, 1898.

In a case seen with the attending physician in June, 1896, a girl of seven had been ill eight weeks with pneumonia and a supposed relapse. I found a pyothorax actually pouting or ready to burst, in the fifth intercostal space, just outside the nipple line. An incision here through the skin was followed by a rush of pus. Resecting an inch of the eighth rib in the posterior axillary line, against the advice of a bystander who thought the child could not survive the operation, she made a good recovery.

The cause of the reprehensible delay in diagnosis in adults suffering from empyema lies in the timidity which prevents an exploratory needle puncture when the physical signs point to fluid in the chest, unless, indeed, the physical signs have been entirely misinterpreted and some other intra- or extrathoracic condition diagnosticated. In July, 1896, I saw a patient who was treated several weeks for abscess of the liver until the exploring needle finally demonstrated pus in the pleural sac at the base of the right chest posteriorly. Signs indicating fluid were present over a small area at the base of the lung and were taken to mean an enlarged liver pushed up into the thorax. Early attempts to find pus with the exploring needle in this region failed because the needle was entered too deeply in the search for the pus of an hepatic abscess. Thoracotomy opened an encapsulated empyema, the floor of which was the diaphragm and the roof the base of the right lung.

In another case, seen in June, 1895, a patient upon whom I made a thoracotomy finding right-sided empyema with the lung compressed into a small space in the region of the root of the lung, had been treated since the preceding April for consolidation of the lung due to tuberculosis. Here the hectic fever and general septic condition and absence of emaciation should have been found incommensurate with the progress of the supposed tubercular process long before, even though the typical physical signs of pleuritic fluid which were present had been so misconstrued.

When the physical signs of fluid in the pleural cavity can be found there is no pathognomonic sign or symptom of pyothorax outside of pus drawn by the aspirating syringe and there is no other way of distinguishing between a serous and purulent exudate. Chills, fever, sweatings, progressive emaciation and anemia are conditions which excite suspicion and point to the probability of pus, but they may be due to disease of tubercular origin or to septic processes elsewhere in the thorax or in the body.

The diagnosis of pyothorax, therefore, embraces the physical signs of fluid in the pleural sac plus the pus which is drawn into the barrel of the exploring aspirating syringe. It may further be said that this statement holds true when the condition of the empyema is lateral, bilateral, sacculated or encapsulated, diaphragmatic or interlobar.

The diagnosis of fluid in the thorax depends upon the signs obtained by the physical examination. While these signs are distinctive, a close study at times is necessary to exclude consolidation of the lung, interthoracic new growths and thickened pleural surfaces, due to former inflammatory conditions.

Inspection of the bared chest may reveal lack of the natural movements of the affected side in breathing, with fulness of the intercostal spaces and bulging during the inspiratory effort. Increased frequency of breathing, with exaggeration of the movements of the sound side, are usually present. The apex impulse of the heart may be displaced especially when the fluid is on the left side. In old cases there occurs curving of the spine with the concavity toward the affected side.

On palpation vocal fremitus is absent over the fluid, or at least is diminished as compared with the sound side, or with the affected side over the compressed lung. Vocal fremitus may be present in the presence of pyothorax when thick pleuritic adhesions communicate vibrations, but under these circumstances it is localised with diminished or absent vocal fremitus surrounding the spot where the adhesions occur.

Auscultation reveals absence of respiratory murmur, except in the presence of a thin layer of fluid. Bronchial or tubular breathing may be heard, transmitted from the distant compressed lung, and inflammatory conditions in such lung tissue will allow of the transmission of râles. Vocal resonance is diminished, but distant bronchophony can be heard when the patient speaks loudly. In children the pronounced and unusual transmission of voice sounds is explained by Dewitt Sherman, of this city, as due to vibration readily conducted along bronchial tubes, consolidated compressed lung and from the thickened visceral to the thickened parietal pleural surfaces. Egophony may be heard over collections of fluid near the lower angle of the scapula. Friction sounds of roughened pleural surfaces occur in the presence of adhesions and even over large collections of fluid may cause the superficial examiner to overlook hydro- or pyothorax.

Percussion produces flatness and resistance to the finger over fluid. Dulness occurs over consolidated lung and thickened pleural surfaces and intrathoracic new growths. Where lobar pneumonia is present, dulness, almost amounting to flatness, is obtained. Increased vocal fremitus distinguishes the case from hydro- and pyothorax. The pathognomonic sign of fluid is a change in the level of flatness on percussion with a change in the position of the patient's body.

When now the physical signs indicate pleuritic effusion the various factors of etiology and history may play their part in determining pyothorax. The history will aid in those cases in which the onset and early progress of the disease present typical signs of pleurisy. There is, of course, no doubt of the diagnosis when the history of perforation of the chest by bullet, knife or other agents can be obtained, or when caries or necrosis of the spine or rib can be shown as cause of septic pleurisy. The history is not so helpful when, following a pneumonia or a pleuro-pneumonia, a subacute pleurisy with effusion develops insidiously. Following or coëxisting with pneumonia, rheumatism, nephritis, the exanthemata, typhoid fever and pyemic conditions, pleurisy is known to develop and a resulting pyothorax may be much obscured. In December, 1897, I was called to the Deaconess's Home to operate in haste on an emeigency case of empyema. I resected a portion of rib, evacuating a large pulmonic abscess, which the physician in attendance on the patient had considered an empyema. She was coughing constantly and spitting a most offensive fetid pus and was intensely septicemic. Air passed freely out of the opening in the thoracic wall from the lung. She lived a few days and was found to have pus in large quantities in the urine. Her history of trouble dated back to September, 1897, when she had her ovaries removed at the Buffalo general hospital. Both contained pus. She did well and left the hospital in two weeks. But within two weeks more she developed a lung trouble which was diagnosticated as pleurisy and she steadily did badly from that time. In another case, seen in June, 1897, at the Deaconess's Home, a pulmonary abscess was diagnosticated following a pneumonia, but thoracotomy revealed a sacculated pyothorax. When the history points to tuberculosis and the tubercle bacillus is discovered in the sputum, a close study of symptoms and signs may be necessary to distinguish between pyothorax and pulmonary abscess or tubercular consolidation of the lung.

Cough, fever and pain are variable quantities in empyema. In a case of pyopneumothorax, seen in December, 1897, cough was present early in the disease, grew less until it nearly ceased and returned with copious expectoration of pus when the empyema opened into a bronchial tube when I resected a rib for this patient. Her temperature was subnormal and had been so for some time. Pain was not present after the early acute process had subsided. Pulse and respiration steadily increased. Fever is usually of the hectic type with evening perspiration more or less profuse.

On several occasions physicians have assured me that patients in whom pyothorax was suspected were not having fever. Finding that their daily visit was made in the morning I suggested leaving a thermometer, instructing some one in the family how to take the temperature. The afternoon and evening temperatures were found ranging between 99° and 102° in several of these cases. Anemia and emaciation become more marked as the case progresses. Chills and sweatings develop as the condition grows more septic and the patient sinks into a condition of septicemia or pyemia. In all uncertain cases the brief delay involved in making a blood count is advised. Leucocytes, if present, add strongly to the signs and symptoms of pyothorax already given.

When now the diagnosis of fluid is certain or even if suspected in doubtful cases, and some or all of the variable septic features of pyothorax are present, the use of the exploring needle must be urged to determine positively the presence and also the nature of the fluid. As to the use of the aspirating syringe for thoracic explorations I feel it can be pronounced a safe measure and it should be oftener employed. There need be no fear of infecting serous or hemorrhagic pleuritic exudate when the needle is inserted with proper precautions. Infection develops from bacteria within the circulation or the respiratory tract by their migration from blood-vessels, bronchial tubes or air vesicles.

As to the procedure to be followed in aspirating the chest I pursue the following course : Sterilise the syringe, which must be in perfect working order, with a needle of the size of a small probe. Thick pus cannot be drawn through too small a needle. Clean the walls of the chest thoroughly by scrubbing with soap and water, then wash off the thoracic wall with corrosive sublimate, 1-1000 solution. Have the patient's arm raised, insert the needle in the seventh or eighth intercostal space in the line of the posterior axillary fold, close to the upper edge of the rib, to avoid the intercostal artery on the lower border of the rib above. To produce local anesthesia, a square inch of the skin where the puncture is to be made should be frozen with chloride of ethyl. Introducing the needle one or two inches, depending upon the thickness of the thoracic wall, the instrument should be steadied and suction made. Withdrawal is effected by a quick movement, and the end of a towel, wet in 1-1000 solution of corrosive sublimate, should be placed over the puncture until an antiseptic compress can be applied and secured by a strip of adhesive plaster. To place a strip of adhesive plaster immediately over the opening is not surgically clean.

If the needle does not enter the pleuritic abscess, or if it be pushed beyond it, or if it enters dense adhesions or extremely thick pleural surfaces, pus may be present and the needle fail to find it. Extremely thick pus may fail to enter the needle if the syringe is not entirely trustworthy, but in expelling the air with the point of the needle held in the palm of the hand a minute drop of pus may be obtained which is sufficient evidence of the presence of pyothorax to justify operation.

The dangers attending exploratory needle puncture of the thorax are three: A serous or hemorrhagic exudate may be infected by an unclean needle. The lung may be lacerated by too deep a puncture and lateral movements of the needle, causing pneumothorax or empyema. Lastly, an improper puncture may injure the intercostal artery, causing hemorrhage.

In conclusion, it may be affirmatively argued that a paper on this subject at this time is opportune. In the changing damp and cold weather of winter and spring, especially in the coming months of March and April, the prevalence of pulmonary and bronchial affections predispose to the development of pyothorax. It behooves the physician, therefore, to be on his guard to detect, as early as possible, the condition of empyema, in order that the benefits of early surgical evacuation of the pleuritic abscess may be obtained.

771 Ellicott Street.

